# Supplementary material for: Long-term consistency in chimpanzee consolation behaviour reflects empathetic personalities
Source: Nat Commun. 2017 Aug 18;8:292. doi: 10.1038/s41467-017-00360-7 (PMC5561193; doi:10.1038/s41467-017-00360-7)

SI GUIDE

File Name: Supplementary Information
Description: Supplementary Figures and Supplementary Tables.

File Name: Peer Review File
Description:

Supplementary Table 1 | Group, sex, age, and kinship of study subjects.

FS1				FS2			
**Subject[1]**	**Sex[2]**	**D.O.B.[3]**	**Age-class[4]**	**Subject[1]**	**Sex[2]**	**D.O.B.[3]**	**Age-class[4]**
Jimoh	M	01/64	A	Phineas	M	01/66	A
Marilyne	F	01/71	A	Amos	M	11/81	A
Reinette	F	12/87	J	Barny	M	05/89	J
Gwennie	F	01/69	A	Chip	M	03/89	J
Socko	M	01/87	J	Magnum	M	07/89	J
Claus	M	12/92	I	Ericka	F	10/73	A
Mai	F	01/64	A	Virginia	F	04/91	J
Natasha	F	12/87	J	Jaimie	F	05/95	I
Missy	F	07/93	I	Tai	F	01/67	A
Borie	F	01/64	A	Daisey	F	10/89	J
Georgia	F	08/80	A	Julianne	F	05/98	I
Kate	F	10/89	I	Barbi	F	06/76	A
Liza	F	03/94	I	Sean	M	03/92	I
Rita	F	09/87	J	Cameron	M	01/95	I
Tara	F	09/95	I	Waga	F	03/82	A
Atlanta	F	08/65	A	Karri	F	01/95	I
Rhett	M	04/89	I	Cynthia	F	06/80	A
Peony	F	01/68	A	Reid	M	07/93	I
Anja	F	01/80	A	Vivienne	F	07/74	A
Bjorn	M	07/88	J	Steward	M	09/93	I
Donna	F	04/90	I	Pollyanna	F	02/89	J
Azalea	F	03/97	I	Sierra	F	07/89	J

[1] Offspring indicated by indentations (e.g., Anja = daughter of Peony; Bjorn = son of Anja)

[2] M = Male; F = Female

[3] Approximate date of birth in month/year

[4] Refers to age-class at onset of study period; I = Infant; J = Juvenile; A = Adult

Supplementary Figure 1 | Developmental curves for grooming and play behavior. These developmental trends illustrate our reasoning for calculating a separate Composite Sociality Index (CSI) for infants/juveniles, which emphasized play behavior rather than grooming (which was used to calculate adolescent/adult CSIs; see switch-points displayed below). Data are shown separately for each group (FS1 and FS2).

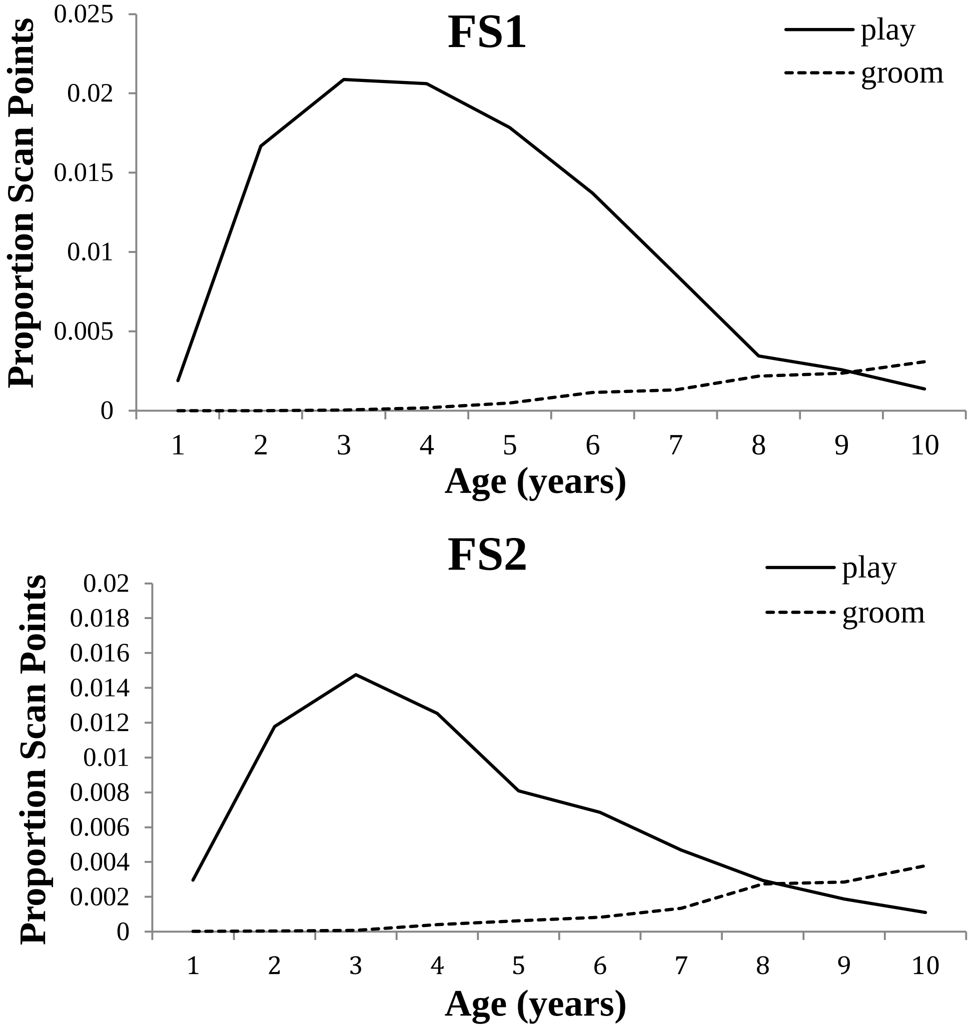

Supplement: Supplementary file 1 — Supplementary Information [file 41467_2017_360_MOESM1_ESM.pdf]
